# Comparative analysis of fetal dose sparing between a C‐arm linac and an O‐ring linac in a SIB‐VMAT sarcoma treatment for a pregnant patient: A technical note and case report

**DOI:** 10.1002/acm2.14556

**Published:** 2024-11-04

**Authors:** Wesley Rivais, Louis Constine, Matthew Pacella, Neil Joyce, Maimuna Nagey, Matthew Webster, Jihyung Yoon, Hyunuk Jung, Sean Tanny, Olga Maria Dona Lemus, Dandan Zheng

**Affiliations:** ^1^ Department of Radiation Oncology University of Rochester Medical Center Rochester New York USA; ^2^ Department of Medical Physics Creighton University Omaha Nebraska USA

**Keywords:** c‐arm, fetal dose, O‐ring, radiotherapy, SIB, VMAT

## Abstract

**Purpose:**

To compare the effect of two linacs designs on fetal dose sparing on a pregnant patient, including estimation of the fetal dose, and the effect of a lead apron.

**Methods:**

A patient with a high‐grade sarcoma located in the right knee/lower thigh was prescribed 51 Gy (1.7 Gy/Fx) with a simultaneous‐integrated‐boost (SIB) of 60 Gy to a smaller volume, starting in the 26th gestational week. Volumetric modulated radiation therapy (VMAT) plans with 6MV‐FFF were developed using identical dosimetric constraints on a Varian Truebeam Edge with HD‐MLC and a Varian Halcyon with double‐stacked MLC. Based on patient dimension measurements, an anthropomorphic phantom was constructed using a Rando phantom and saline bags in the patient's Vac‐Lok bag. Phantom measurements were performed using OSLDs and TLDs placed at three different planes, corresponding to the pubis, the umbilicus, and the fundus based on patient measurements and projected gestational age, to estimate the fetal dose. Three experimental scenarios were measured, each with CBCT‐based image guidance for an accurate, reproducible setup: Edge, Halcyon, and Halcyon with a tri‐folded lead apron (0.5 mm × 3 = 1.5 mm Pb) over the phantom abdomen.

**Results:**

Plan quality and total MUs are comparable between the Edge and Halcyon plans. The OSLD‐measured whole‐course dose to the pubis, umbilicus, and fundus were 18.8, 13.1, and 11.7 cGy, respectively, on Halcyon, on average 27.8% lower than Edge. The repeatability within either dosimeter was good, although TLD showed systematically lower doses. Importantly, both dosimetry systems showed a lower measured fetal dose for the Halcyon plan compared with the Edge plan. Adding a tri‐folded lead apron over the abdomen did not meaningfully lower the measured dose.

**Conclusion:**

In this case study, Halcyon demonstrated a better sparing of out‐of‐field fetal dose compared to TrueBeam Edge. It was shown that lead aprons do not provide additional fetal dose sparing.

## 1 INTRODUCTION

In radiation therapy for pregnant patients, fetal dose estimation and sparing are crucial to minimize the risk to the developing fetus. When delivering radiation therapy to pregnant patients, fetal exposures can cause serious risks to the developing fetus, such as growth retardation, congenital abnormalities, and an increased risk of cancer in later life.^1^, ^2^, ^3^ Higher radiation doses are associated with a greater likelihood and severity of fetal risks with some established dose thresholds.^2^, ^4^ These risks also vary in severity according to the gestational age of the fetus, with a 2.5‐fold increased risk in the first trimester compared to the second and third trimesters.^5^ Therefore, it is important to precisely estimate the fetal dose to guide clinical decision‐making, provide informed consent to the patient, and devise an appropriate course of action that minimizes risks to the fetus while effectively treating the mother's cancer. Following the widely adopted method of using anthropomorphic phantoms for these types of dose assessments^6^, ^7^, ^8^, ^9^ and effectively using readily available materials in the clinic to implement a hybrid “custom” anthropomorphic phantom, this case report focuses on a pregnant patient undergoing radiation therapy for a sarcoma in the right leg, comparing the fetal dose sparing capabilities of two linear accelerators (linacs) systems: Varian Truebeam Edge and Varian Halcyon (Varian Medical Systems, Palo Alto, CA). The treatment was designed using volumetric modulated radiation therapy (VMAT) with a simultaneous integrated boost (SIB).

The two compared linacs are both widely used in radiation therapy around the world. The TrueBeam model has been a workhorse model for over a decade with the versatility to deliver a wide range of radiation treatments such as stereotactic radiosurgery (SRS), stereotactic body radiotherapy (SBRT), intensity‐modulated radiation therapy (IMRT), VMAT, and electron therapy. It also allows treatments with non‐coplanar beams, or at extended source‐to‐surface distances (SSD). TrueBeam uses a conventional C‐arm gantry design with a single layer of multi‐leaf collimators (MLC) that have either a regular width of 5 mm central and 10 mm peripheral, or a high‐definition width of 2.5 mm central and 5 mm peripheral. The latter is used in TrueBeam Edge. In comparison, Halcyon is a more recent design, featuring a ring gantry and a single energy of 6 MV flattening‐filter‐free (FFF) photons. It is designed for streamlined workflow and efficiency, with fast imaging and IMRT/VMAT delivery, to provide higher patient throughput. Halcyon has a double‐stacked MLC, each with a 10 mm width, and does not allow couch rotation due to the ring gantry design. The MLC thickness/height was 67 mm for TrueBeam Edge and 77 mm for Halcyon, respectively. In addition to distinct MLC designs, TrueBeam and Halcyon also have different collimator designs and other geometric differences in the gantry head. In TrueBeam linacs, independent jaws provide secondary collimation and MLC provides tertiary collimation. In contrast, Halcyon has a jawless gantry in which the upper layer MLC rides along the lower layer MLC to provide back‐up shielding like the jaws. Also, Halcyon uses an integrated beam stopper to eliminate the need for primary room shielding but TrueBeam linacs do not use beam stoppers. All these design differences could contribute to different linac head leakage and head scatter, and hence different out‐of‐field doses.^10^, ^11^, ^12^ As these two linacs systems have major design differences and are both available in our clinic, for the case to be presented here, we aimed to test how the two linacs would perform in terms of fetal dose sparing or if one system would outperform the other.

## 2 CASE PRESENTATION

The subject of this study is a 38‐year‐old pregnant female diagnosed with soft‐tissue sarcoma at the right knee/distal thigh. The patient was simulated with a lower‐half‐body Vac‐Lok bag (CIVCO Medical Solutions, Kalona, IA) in a feet‐first supine position with a CT scan from the mid‐thigh to the ankle. Measurements were also taken for the cranial‐caudal distances between the simulation reference point and the 3 reference points for fetal dose estimation: the fundus, symphysis pubis, and umbilicus. The actual patient treatment commenced in the 26th gestational week.

## 3 METHODS AND MATERIALS

Treatment planning was carried out using the Eclipse planning system v15.6 (Varian Medical Systems, Palo Alto, CA). The Acuros External Beam (AXB) algorithm was used for dose calculation. Treatment planning was carried out following dose constraints defined in RTOG 0630 for extremity sarcoma.^13^ For this comparative study, VMAT plans using 3 partial‐arc 6MV FFF beams were generated for both TrueBeam Edge and Halcyon 6 (Varian Medical Systems, Palo Alto, CA) using identical beam setup and identical optimization constraints and objectives. The isocenter was placed as superiorly as the field size would allow to minimize the beam divergence superiorly to the abdominal region, and was kept identical for the two plans. No other deliberate steps were taken during treatment planning to reduce the out‐of‐field dose. The treatment prescribed a dose of 51 Gy/30 fractions (1.7 Gy per fraction) to the planning target volume (PTV) PTV_5100, with a simultaneous‐integrated boost (SIB) of 60 Gy (2 Gy per fraction) to a smaller volume PTV_6000.

To estimate the fetal dose, a customized anthropomorphic phantom mimicking the patient's anatomy was constructed using a Rando phantom (Alderson Research Laboratories, Stanford, CT) for the head and torso, and saline bags within the patient Vac‐Lok bag for the legs. Based on the thigh, knee, and leg girth physically measured on the patient, the size, orientation, and number of saline bags were fitted to reasonably best replicate the patient's body habitus. Dosimetric measurements were conducted using optically stimulated luminescent dosimeters (OSLDs) and thermoluminescent dosimeters (TLDs) at the pubis, the umbilicus, and the fundus at measurement depths 10–12 cm depending on the respective craniocaudal location. NanoDots (Landauer, Inc., Glenwood, IL) and the “Radiation Monitoring by Mail” service (University of Wisconsin, Madison) were used for OSLD and TLD measurements, respectively. A Rando phantom slab was chosen for each of these three reference points, with the cranial‐caudal distance between the slab and the isocenter matching the measured distance on the actual patient. Two OSLDs and two TLDs were inserted for each slab. The OSLD readings were collected from a microStar reader (Landauer, Inc., Glenwood, IL) following the vendor's readout procedure. Our existing batch linear calibration curve and vendor‐supplied sensitivity correction factors were used for these single‐use OSLDs. Specifically, a Cs‐137 calibration set and daily QC were used for the reader calibration, an existing low‐dose linear calibration dose‐response curve irradiated with a 6MV primary beam on Edge to 0, 5, 10, and 15 cGy was used for dose calibration, and the vendor‐supplied relative‐sensitivity factor was used for each dosimeter. We used these OSLDs as single‐use dosimeters without bleaching for repeated uses. For TLD calibration, additional calibration TLDs were irradiated with a standard square field solid water setup on Edge using the 6FFF primary beam to deliver 1, 5, and 10 cGy, respectively, each with 3 TLDs. No separate calibration measurements on Halcyon were performed.

Measurements were taken for three different deliveries: using the Halcyon, the Edge, and the Halcyon with a lead apron, with all setups employing CBCT‐based IGRT for precision and reproducibility. The lead apron was a conventional diagnostic imaging lead apron. It had a 0.5 mm lead and was folded three times to cover the phantom abdomen, with a 1.5 mm lead thickness. While the limited thickness of lead was not expected to provide meaningful shielding, the lead apron was included in the phantom measurement test as such lead aprons are used in some clinics for fetal shielding during radiotherapy.^14^ No in vivo measurements on the patient were performed.

## 4 RESULTS

**FIGURE 1:**
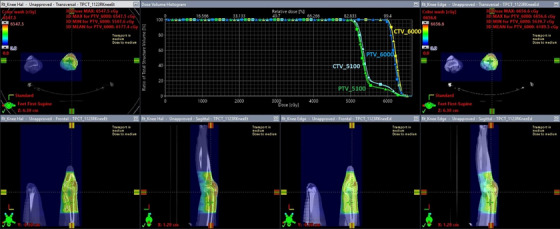
Plan comparison between the Halcyon and the TrueBeam Edge plans on the patient. On the DVH, squares denote the Halcyon plan and triangles denote the Edge plan.

The 3D dose distribution and dose volume histograms (DVH) are displayed in Figure 1 which compares the Halcyon and the TrueBeam Edge plans. Developed with identical beam setups and identical dose constraints and objectives for optimization, the two plans resulted in similar dose distributions around the targets. Distant areas, such as the distal ipsilateral leg, appear to receive lower doses from the Halcyon plan compared with the TrueBeam Edge plan. Table 1 lists the detailed beam parameters for the two plans. Both plans used VMAT with three partial arcs on the patient's right side. Optimized with identical optimization parameters, the Edge plan had a total monitor unit (MU) 11% higher than the Halcyon plan (670 vs. 601.5 MU). With the high‐dose SIB target prescribed at 200 cGy per fraction, the Halcyon and Edge plans corresponded to a modulation factor of 3.01 and 3.35, respectively, defined as the ratio of MU and prescribed dose.

**TABLE 1:** Beam parameters of the VMAT plans for the two linacs.

	Gantry rotation	MU‐Halcyon	MU‐Edge	MU difference
Arc‐1	300 CW 179	209.8	254	44.2
Arc‐2	179 CCW 300	197.9	222	24.1
Arc‐3	300 CW 179	193.8	194	0.2
Total MU		601.5	670	68.5

**FIGURE 2:**
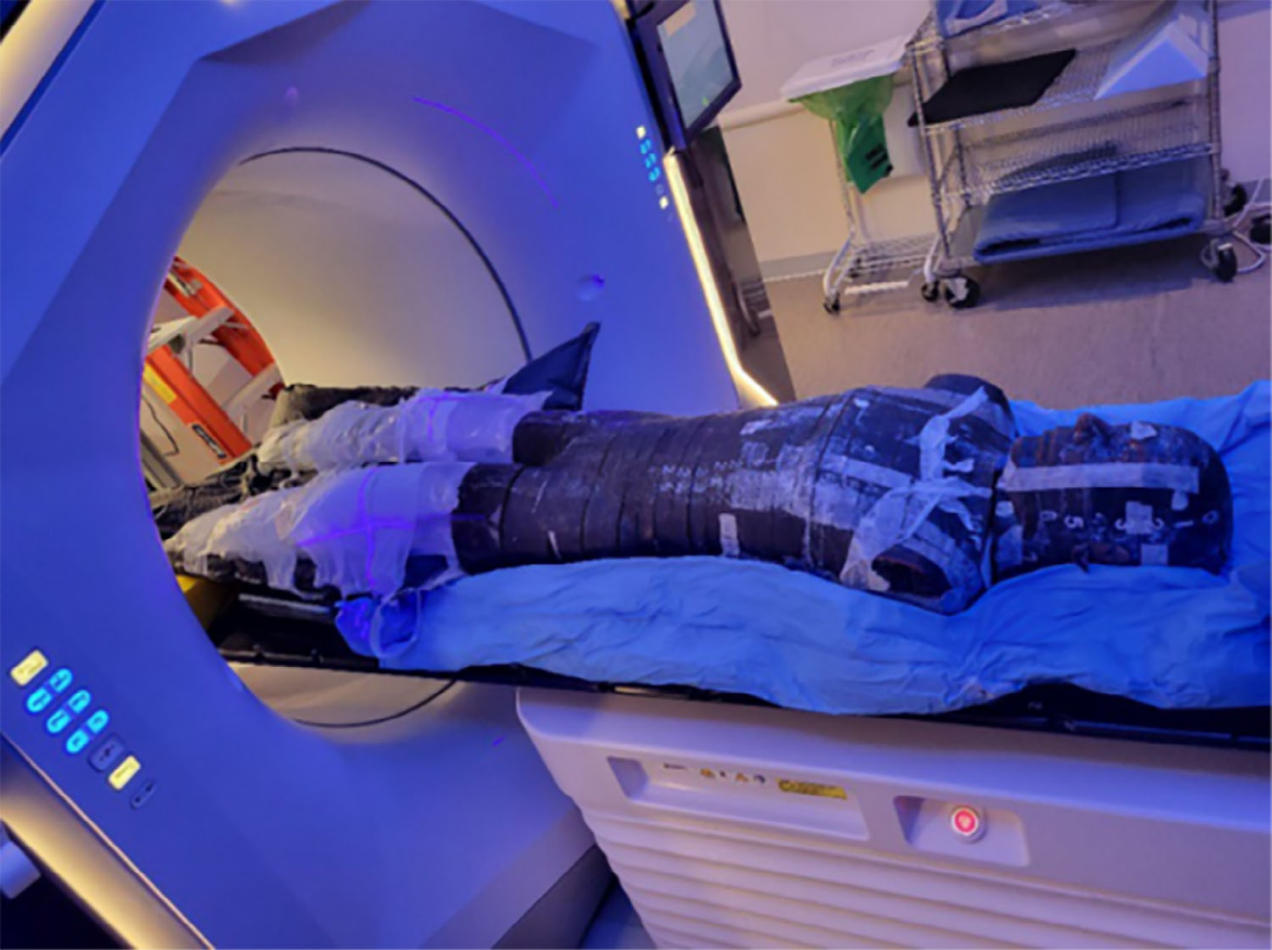
Based on patient measurements of leg dimensions and isocenter‐to‐reference‐point distances, an anthropomorphic phantom was constructed using a Rando phantom and saline bags in the patient Vac‐Lok bag (black). Pictured was the phantom positioned on Halcyon during setup.

The phantom as shown in Figure 2 was successfully constructed to mimic the patient. The patient Vak‐Lock bag was used to reproduce and position the phantom legs made of saline bags, and a separate immobilization bag was made for the Rando phantom in the upper body.

**FIGURE 3:**
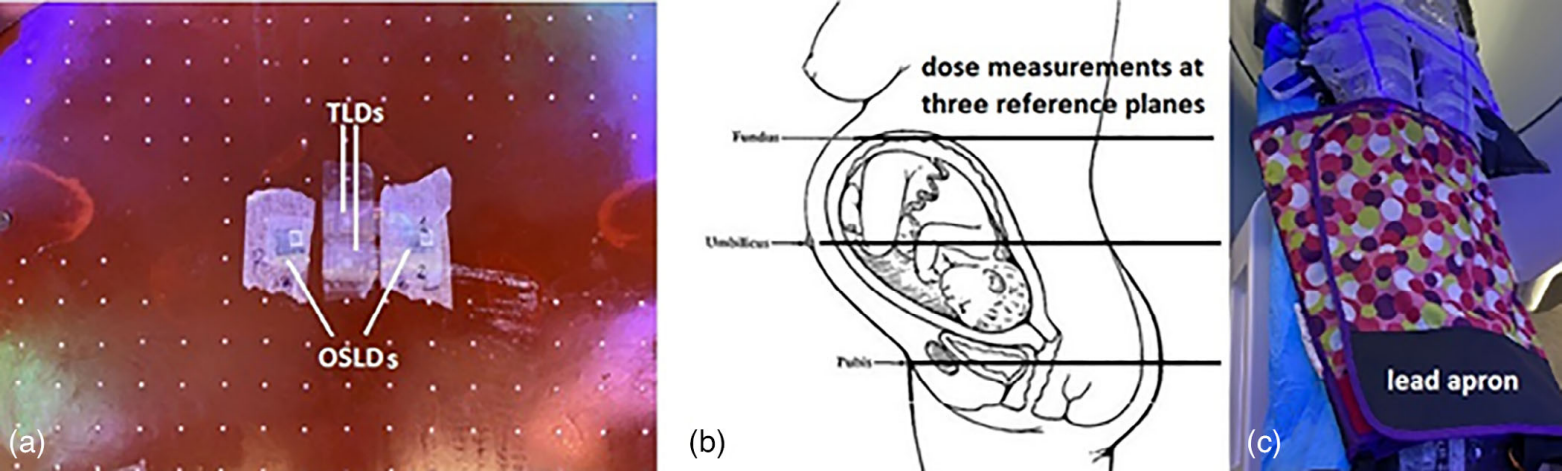
Two OSLDs and two TLDs (Panel a) are placed at each of the three reference planes (pictured in Panel b) representing the pubis, the umbilicus, and the fundus points, respectively. For Halcyon delivery, a lead apron for diagnostic imaging use was folded three times and placed on the phantom abdomen to assess its dose sparing effect (Panel c).

**FIGURE 4:**
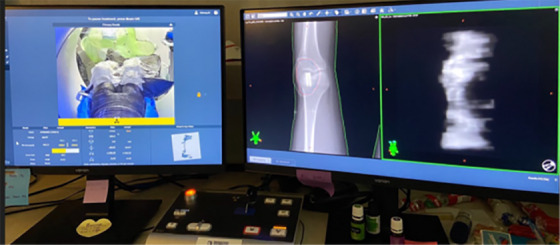
A console computer view during the Halcyon delivery. Customized phantom realistically mimics the patient geometry. Careful phantom positioning and CBCT guidance ensured accurate and reproducible phantom setup across experiments.

With distances from the patient measurements, three Rando slabs were selected to place dosimeters for dose measurements at the three reference points. The three reference points were the pubis, the umbilicus, and the fundus reference points as defined by the Task Group Report 36 (TG36) of the American Association of Physicists in Medicine,^4^ as shown in Figure 3b. On each slab, two OSLDs and two TLDs were placed for measurements, as shown in Figure 3a. Measurements were made at three deliveries: Halcyon, TrueBeam Edge, and Halcyon with a tri‐fold lead apron on the phantom abdomen (shown in Figure 3b). Careful phantom positioning and cone beam CT (CBCT)‐based imaging guidance were performed at each delivery to ensure an accurate and reproducible experimental setup. Fiducial marks on the bags were used for setup with corresponding couch shifts applied. Additional fiducial marking of the isocenter on the phantom was confirmed on CBCT images. Figure 4 shows a screen capture at the Halcyon console, with a camera view of the phantom on the left and the patient digitally reconstructed radiography (DRR) with cine MV image during the phantom delivery on the right.

Dosimetric results were summarized in Table 2 for OSLD‐measured results, and in Table 3 for TLD‐measured results. Both the OSLD and the TLD measurements showed good repeatability based on the close agreements of the two detectors of the same kind on each plane of each measurement. However, the OSLD‐measured dose values were systematically higher than the TLD‐measured values. Comparing the two linacs, Halcyon consistently resulted in lower OSLD‐measured doses across all planes compared to Edge, with reductions of about 27.8% on average. TLD measurements corroborated these findings, indicating a similar trend, but the absolute measured dose values were systematically lower. The introduction of a tri‐folded lead apron (1.5 mm Pb) did not substantially affect the fetal dose.

**TABLE 2:** Cranial‐caudal distances to the superior field edge and OSLD measurement results. The total dose over the entire course is reported using the mean fractional dose reading multiplied by 30 fractions. The fractional dose is also listed in the parenthesis using the raw measurement results from the two OSLDs to give an appreciation of precision and repeatability.

	Distance to sup field edge (cm)	Halcyon w/o Pb (cGy) Total (fractional)	Halcyon w/ Pb (cGy) Total (fractional)	Edge (cGy) Total (fractional)
Fundus	46	11.7 (0.370, 0.409)	11.6 (0.389, 0.384)	14.5 (0.475, 0.491)
Umbilicus	38	13.1 (0.441, 0.435)	12.8 (0.413, 0.443)	16.1 (0.518, 0.553)
Pubis	23	18.8 (0.638, 0.615)	18.2 (0.581, 0.631)	25.8 (0.852, 0.865)

**TABLE 3:** TLD measurement results. The total dose over the entire course is reported using the mean fractional dose reading multiplied by 30 fractions. The fractional dose is also listed in the parenthesis using the raw measurement results from the two TLDs to give an appreciation of precision and repeatability.

	Halcyon w/o Pb (cGy) Total (fractional)	Halcyon w/ Pb (cGy) Total (fractional)	Edge (cGy) Total (fractional)
Fundus	3 (0.1, 0.1)	3 (0.1, 0.1)	6 (0.2, 0.2)
Umbilicus	3 (0.1, 0.1)	4.5 (0.1, 0.2)	7.5 (0.2, 0.3)
Pubis	9 (0.3, 0.3)	9 (0.3, 0.3)	18 (0.6, 0.6)

## 5 DISCUSSION

This work showcased the use of a customized anthropomorphic phantom for out‐of‐field measurements prior to patient treatment for fetal dose estimation on a pregnant patient receiving radiation therapy. As they are both available in our clinic, two popular linac systems with distinct designs were examined to see if one has a greater advantage over the other in terms of minimizing out‐of‐field radiation. The customized anthropomorphic phantom reproduced the patient case reasonably well for these tests, and CBCT image guidance was employed to ensure the accurate setup for these phantom measurements. This project answered a clinically relevant question in a unique manner and similar cases in the future could be handled the same.

The Halcyon system demonstrated superior performance in sparing the fetus from radiation exposure than the TrueBeam Edge system. The advantage was likely due to the double‐stacked MLC design for Halcyon and other design differences between the two systems, which reduced head leakage and head scatter, leading to the reduced out‐of‐field dose. The single beam energy with FFF available on Halcyon might not have been ideal for cases prescribed with simple 2D treatments, such as using parallel opposed uniform beams. However, for the presented case with SIB‐VMAT, the FFF beam carried the additional advantage of reduced scatter from not using a flattening filter.

A lead apron for diagnostic imaging, even folded three times, did not provide meaningful additional shielding. Because the estimated fetal dose was acceptable (<50 cGy) even based on the higher OSLD‐measurement values, no additional heavy‐duty shields with several inches of lead equivalent attenuation were attempted, such as with a “bridge over patient,” “table over treatment couch,” or “mobile shields” design described in TG36^6^. Should such a heavy‐duty shield be needed, the ring gantry design of Halcyon may pose potential clearance problems while the open C‐arm design of TrueBeam may be more amenable.

Our study used two types of luminescent dosimeters, OSLD and TLD, for the phantom measurements. In addition to being readily available in clinics for the convenient multi‐point patient and phantom measurements, these dosimeters also have a relatively high sensitivity and precision in detecting low dose levels and were used in numerous prior studies for these types of dose measurements.^15^, ^16^, ^17^, ^18^, ^19^, ^20^, ^21^, ^22^ Notably, the OSLD vendor recently issued dosimeter and reader recalls, which could raise questions about the validity of our OSLD measurement results. On the other hand, the same OSLD system (the batch of OSLDs used in the study was purchased in 2021) has been used for over a decade in our clinic, as has been in many other clinics around the world and was deemed with good accuracy and validity in numerous published studies.^1^, ^17^, ^19^, ^22^, ^23^, ^24^, ^25^, ^26^, ^27^, ^28^, ^29^ Largely due to the recall, in this study we also adopted a second dosimetry system, TLD through the University of Wisconsin's “Radiation Monitoring by Mail”. Nevertheless, there are many inherent uncertainties associated with out‐of‐field dose measurements with these dosimeters, especially at low doses. Though more water‐equivalent compared with some other alternative dosimeters like diode and metal‐oxide‐semiconductor field‐effect transistor (MOSFET), Al_2_O_3_ (OSLD) and LiF (TLD) both still have slightly higher effective atomic numbers than that of water, hence leading to overresponses to radiation at lower energies. Due to the higher effective atomic number, OSLD would have more overresponses at low energies than TLD, which could have contributed to the higher OSLD readings in our experiments. Though both dosimeters have a vendor‐suggested dose operating range much lower than our measurements, the dose‐response of OSLD and TLD is not perfectly linear, with a supralinearity reported at the higher dose range.^25^, ^30^, ^31^, ^32^ In addition, both detectors also have other uncertainties such as beam angular dependence, signal fading, and detector sensitivity. Although they were not explicitly studied in our case study, these uncertainties need to be considered in understanding our results. Previous studies have reported an uncertainty within 2% for these dosimeters when explicitly correcting for these uncertainty sources.^23^, ^26^ When uncorrected, these uncertainties can be of magnitudes in 2%–6% relevant to our experiments.^16^, ^23^, ^24^, ^25^, ^26^, ^27^, ^29^, ^30^, ^31^, ^32^, ^33^

Between the two dosimetry systems used in this case study, the TLD readings were noticeably lower than the corresponding OSLD readings. We speculate that the systematic discrepancy stemmed from the dosimeter precisions, our calibrations, and the multi‐factored uncertainties of these dosimeters and our experiments. Although both dosimeters have relatively good energy independence and dose linearity, residual uncertainties exist, especially for the low signal levels and beam energy spectrums for our out‐of‐field dose measurements. The TLDs were calibrated at dose levels of 1, 5, and 10 cGy using the in‐field primary beam, while the single‐fraction phantom TLD readings ended up being in the range of 0.1–0.6 cGy, leading to a higher uncertainty due to extrapolation. Linacs are also known to exhibit higher MU linearity uncertainties when delivering less than 5 MUs, and the uncertainty can be up to 5% for 1 MU. This would contribute to the uncertainties of calibration, and hence the measurements. In retrospect, calibration could have been delivered with alternative settings that would allow more MUs to deliver the same dose, thereby mitigating this uncertainty. Similarly, the calibration could have been extended in this manner to lower dose levels than the measurement signal levels so that the experimental measurements could use interpolation instead of extrapolation to reduce calibration uncertainty. In addition, to mitigate low signal levels of the experiments, the fetal dose measurements on the phantom could be performed by delivering multiple fractions to build up the signal. Nevertheless, the two dosimetry systems showed similar trends comparing Halcyon and Edge, and the precision and repeatability within either dosimetry system from the pair detectors were reasonably good, as shown in Tables 2 and 3. While the uncertainties could affect the absolute dose values determined by our measurements, our results are useful and definitive for the relative comparison between the two machines.

In addition, AAPM TG158 also provided a comprehensive guide on estimating and measuring the out‐of‐field dose from external beam radiotherapy and recommended dose reduction strategies.^28^ Using the out‐of‐field dose data from 9 IMRT studies summarized in that report, our three measurement points would have received 1.6–18, 2.1–24, and 5.4–39 cGy, respectively. While many factors led to the notable spread in these published data, such as differences in target volumes, modulation, beam energy, machine type, and delivery technique, both our OSLD and TLD measurement results fall in line with these previous findings.^34^, ^35^, ^36^, ^37^, ^38^ The OSLD measurements were used for fetal dose estimation and clinical decision‐making, as its higher readings provided a more conservative estimation of fetal dose. Based on the phantom test results, the patient in our case was treated on Halcyon, without a lead apron. The estimated 14.5 cGy average dose to the fetus over the gestational age of 26–32 weeks belongs to the category where, in comparison to earlier stages of pregnancy, noncancer health consequences are comparatively less common. According to the prenatal radiation exposure fact sheet,^2^ doses less than 50 cGy (50 rads) typically do not result in significant noncancer health impacts throughout the 26–38 week fetogenesis stage. Nonetheless, there may still be certain risks connected to the dose range of 5 to 50 cGy (5–50 rads) to which our patient's fetus has a small risk. Possible side effects may include a minimal risk for growth retardation and IQ loss, and an observable increased lifetime cancer risk. While possible, growth retardation and IQ loss are more likely to be problematic at doses greater than 50 cGy (50 rads). In our case, 14.5 cGy dose falls below this threshold. For cancer risk, the fact sheet^2^ indicates that the risk of cancer can increase from 1% to 6% above the baseline risk between 5 and 50 cGy. This suggests that there may be an increased chance of developing cancer. As a result, even while the increased risk is minimal, it is nonetheless higher than in people who have not had any additional radiation exposure.

## 6 CONCLUSION

A case study is presented where fetal dose estimation was conducted based on phantom measurements for radiation therapy of a pregnant patient with knee sarcoma. The customized anthropomorphic phantom aided the investigational comparison between two linacs systems with distinct designs and demonstrated the out‐of‐field fetal dose‐sparing advantages of Halcyon over TrueBeam in our case.

## AUTHOR CONTRIBUTIONS

Wesley Rivais: Methodology, investigation, data collection and analysis, and writing the original draft. Louis Constine: Methodology and writing the original draft. Matthew Pacella: Methodology, investigation, data collection, and analysis. Neil Joyce: Methodology and investigation. Maimuna Nagey: Data collection and analysis. Matthew Webster, Jihyung Yoon, Hyunuk Jung, Sean Tanny, and Olga Maria Dona Lemus: Methodology and investigation. Dandan Zheng: Conceptualization, methodology, investigation, data collection and analysis, and writing the original draft. All authors contributed to the review & editing of the manuscript.

## CONFLICT OF INTEREST STATEMENT

The authors declare no conflicts of interest.
